# Motor Imagery in Unipolar Major Depression

**DOI:** 10.3389/fnbeh.2014.00413

**Published:** 2014-12-04

**Authors:** Djamila Bennabi, Julie Monnin, Emmanuel Haffen, Nicolas Carvalho, Pierre Vandel, Thierry Pozzo, Charalambos Papaxanthis

**Affiliations:** ^1^Department of Clinical Psychiatry, University Hospital of Besançon, Besançon, France; ^2^EA 481 Neurosciences, University Hospital of Besançon, Besançon, France; ^3^FondaMental Foundation, Créteil, France; ^4^Centre d’Investigation Clinique en Innovation Technologique de Besançon (CIC-IT 808) INSERM, Besançon, France; ^5^FHU Integrated Center for Research in Inflammatory Diseases (InCREASe) INSERM, Besançon, France; ^6^UFR STAPS, Université de Bourgogne, Dijon, France; ^7^Unité 1093, Cognition, Action et Plasticité Sensorimotrice, INSERM, Dijon, France; ^8^Robotics, Brain and Cognitive Sciences Department, Istituto Italiano di Tecnologia, Genoa, Italy; ^9^Institut Universitaire de France (IUF), Dijon, France

**Keywords:** major depressive disorder, psychomotor retardation, motor imagery, mental chronometry, movement speed, speed/accuracy trade off

## Abstract

**Background:** Motor imagery is a potential tool to investigate action representation, as it can provide insights into the processes of action planning and preparation. Recent studies suggest that depressed patients present specific impairment in mental rotation. The present study was designed to investigate the influence of unipolar depression on motor imagery ability.

**Methods:** Fourteen right-handed patients meeting DSM-IV criteria for unipolar depression were compared to 14 matched healthy controls. Imagery ability was accessed by the timing correspondence between executed and imagined movements during a pointing task, involving strong spatiotemporal constraints (speed/accuracy trade-off paradigm).

**Results:** Compared to controls, depressed patients showed marked motor slowing on both actual and imagined movements. Furthermore, we observed greater temporal discrepancies between actual and mental movements in depressed patients than in healthy controls. Lastly, depressed patients modulated, to some extent, mental movement durations according to the difficulty of the task, but this modulation was not as strong as that of healthy subjects.

**Conclusion:** These results suggest that unipolar depression significantly affects the higher stages of action planning and point out a selective decline of motor prediction.

## Introduction

Motor imagery is as a mental process during which a specific action is internally simulated without any overt motor output. According to the simulation theory, mental actions are motor actions that are not overtly executed (Jeannerod and Decety, 1995; Jeannerod, 2001). Numerous studies have addressed the behavioral and cerebral correlates of motor imagery, and its relationship with motor planning and execution. It has been reported that actual and mental actions follow the same motor rules (e.g., speed–accuracy trade-off, speed-curvature relationship) and retain the same temporal structure (Decety and Jeannerod, 1995; Maruff et al., 1999; Bakker et al., 2007; Gueugneau et al., 2008; Papaxanthis et al., 2012). Furthermore, mental training improves motor performance (Yaguez et al., 1998; Gentili et al., 2006, 2010; Allami et al., 2008; Avanzino et al., 2009) and enhances muscular force (Yue and Cole, 1992; Zijdewind et al., 2003; Ranganathan et al., 2004). Lastly, neuroimaging studies revealed a common activation of specific brain regions recruited during both motor imagery and motor production, pointing to the parietal and prefrontal cortices, the supplementary motor area, the premotor and primary motor cortices, the basal ganglia, and the cerebellum (Lotze et al., 1999; Jeannerod, 2001; Guillot and Collet, 2005; Szameitat et al., 2007; Munzert et al., 2009; Hetu et al., 2013).

Experimental paradigms using motor imagery offer a useful and sensitive behavioral tool to investigate the unconscious process of action representation (Jeannerod and Decety, 1995). Motor imagery has been extensively used to gain insight into the action system of both healthy and diseased populations. The advantage of motor imagery is that one can explore the internal processes of action planning and preparation, while avoiding sensory and motor confounds related to motor execution. This feature is especially important when studying motor impairments in clinical populations, like those in neuropsychiatric and neurological syndromes, in which motor execution is impaired or even absent. In these cases, the ability or inability of generating motor images can indicate whether the planning stage of an action is intact or impaired, respectively. Motor imagery impairments have been found in Parkinson disease (Dominey et al., 1995; Helmich et al., 2007; Heremans et al., 2011), in cerebellum syndrome (Kagerer et al., 1998), after lesions in motor and parietal cortex (Sirigu et al., 1996; Danckert et al., 2002; Malouin et al., 2004), in neglect patients (Coslett, 1998), in patients with fatigue syndrome (de Lange et al., 2004), and in multiple sclerosis (Heremans et al., 2012; Tacchino et al., 2013).

Psychomotor retardation (PMR) is a central feature of depression that can have clinical and therapeutic implications, and may severely impact on patient’s psychosocial functioning. PMR modifies all the actions of the individual, including motility, mental activity, and speech (Widlocher, 1983). Clinical and experimental studies, questioning the motor aspects of PMR, have enhanced the comprehension of important pathophysiological mechanisms in depression (Sobin and Sackeim, 1997). Nevertheless, cognitive deficits related to PMR are poorly understood. Several studies have drawn similarities between bradyphrenia in depressed patients and bradykinesia in Parkinson disease, specifically in self-initiated movement in reliance to external or internal cues, or in programing the velocity of movement (Caligiuri and Ellwanger, 2000; Rogers et al., 2000). These authors have reported that some aspects of motor deficits are equally present in the two pathologies, and consequently have suggested the possibility that the two phenomena may share some common underlying pathology (Caligiuri and Ellwanger, 2000; Rogers et al., 2000). The basal ganglia system constitutes, therefore, a possible candidate as a site of motor dysfunction common to these two disorders. In addition, PMR was linked to structural alterations in the prefrontal dorsolateral cortex (DLPFC) and hypodopaminergic states of the basal ganglia (Bench et al., 1993; Martinot et al., 2001; Walther et al., 2012). Investigation of motor imagery can strongly contribute to explore the higher stages of action organization underlying PMR in depression.

In the current study, we evaluated the effect of depression on motor imagery ability. Our general aim was to examine whether cognitive aspects of motor function, like action representation and prediction, are affected by major depression disorder (MDD). A group of patients with MDD and an aged-match control group carried out actual and mental arm movements involving strong spatiotemporal constraints (speed/accuracy trade-off paradigm). We recorded actual and mental movement times and used the degree of their similarities (i.e., isochrony) as an indicator of the accuracy of motor imagery/prediction process (Sirigu et al., 1996; Personnier et al., 2010b; Demougeot and Papaxanthis, 2011). Based on the previous literature, we expected patients with MDD to be slower than healthy controls in both executed and imagined movements. We also anticipated a specific decline in motor imagery ability in patients with MDD; that is, significant temporal differences between actual and mental movements. Such impairments could provide an objective marker of brain dysfunction in depression, which impacts the higher stages of motor planning and production.

## Materials and Methods

### Participants

Fourteen patients (eight females, six males, mean 52.7 ± 16.7 years), meeting diagnostic and statistical manual of mental disorders (DSM-IV) criteria for unipolar depression, and 14 healthy adults (seven females, seven males, mean 57.6 ± 11.2 years), matched for age, sex, and education, participated in this study. All of them had normal or corrected-to-normal vision and were right handers (Olfield, 1971). Patients were recruited from the psychiatric wards of the university hospital of Besançon (France). They were included into the study if their score was more than 25 on the Montgomery–Asberg Depression rating Scale (MADRS) (Montgomery and Asberg, 1979) and if they were considered at least stage II treatment resistant (Thase et al., 1995; Rush et al., 2003). Exclusion criteria were: bipolar depression, psychotic features, neurological disease, severe organic disease, and intake of first-generation antipsychotics (FGA). Every patient received an antidepressant medication with escitalopram in a constant dosage (10–20 mg/day) over 4 weeks prior to the experiment. Two patients received substances for augmentation, six patients received second-generation antipsychotics (SGA), and six were treated with benzodiazepines. Participants of the control group were free from any neurological, cognitive, and muscular impairment. They were recruited from the University’s and Hospital’s staff, as well as from the local community. All participants gave written informed consent to participate in the study. Research protocol was approved by the Committee of Protection of Persons (CPP-Est-II), and was conducted in accordance with the principle laid down by the declaration of Helsinski.

### Psychiatric assessment

All patients completed the Montgomery–Asberg Depression Rating Scale (MADRS) (Montgomery and Asberg, 1979), the 24-items Hamilton Depression Rating Scale (HDRS) (Hamilton, 1960), and the Salpetriere Retardation Rating Scale (SRRS) (Widlocher, 1983) to determine the intensity of depression and the clinical severity of retardation. In addition, patients and controls completed the Beck Depression Inventory (BDI; Beck et al., 1961). All patients were severely depressed and showed a marked degree of retardation (see Table 1). The BDI scores in the depressed patients were significantly higher than those in healthy subjects (*Z* = 5.84, *P* < 0.0001).

**Table 1:** **Average (±SD) scores in clinical tests for the major depression disorders (MDD) and the control groups**.

Tests	MDD group (*n* = 14)	Control group (*n* = 14)
MADRS	30.6 ± 4.8	–
HDRS	22 ± 3.9	–
SRRS	23.6 ± 9.9	–
BDI	17.8 ± 4.0	2.13 ± 3.9

*MDD, major depressive disorder; MADRS, Montgomery–Asberg Depression Rating Scale; HDRS, Hamilton Depression Rating Scale; SRRS, Salpetrière Retardation Rating Scale; BDI, Beck Depression Inventory*.

### Experimental design

**Figure 1:**
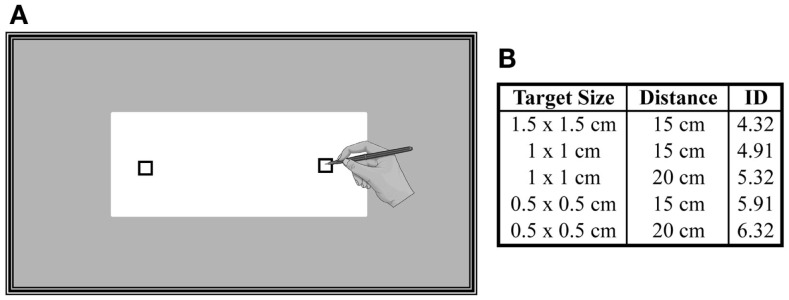
**(A)** Schematic representation of the experimental setup. A sheet of paper (A4 format) was placed on a table and participants had to actually point or to imagine pointing between the targets as accurate and as fast as possible. **(B)** Three different sizes of targets and two inter-target distances were used to modulate the difficulty of the task (ID).

The experiment took place in a quiet room inside the hospital. In order to limit the influence of circadian rhythms on motor and mental performances (Gueugneau et al., 2009; Gueugneau and Papaxanthis, 2010), all experiments were carried in the morning (between 9 and 11 a.m.). Participants were comfortably seated on an adjustable chair in front of a table whose edge was aligned with their chest at the level of the diaphragm. In the middle of the table, a block of paper (A4 format) was placed at a distance of 20 cm from participants’ chest (Figure 1A). In each sheet, two targets were printed (black squares). We used three different sizes of targets (0.5 cm × 0.5 cm, 1 cm × 1 cm, 1.5 cm × 1.5 cm) and two inter-target distances (15 and 20 cm) to modulate the difficulty of the task according to the Fitts’s law (Fitts, 1954):

(1) $$\mathrm{ID}=\log_{2}\left(2A∕W\right),$$

where, ID is the index of difficulty, *A* is the inter-target distance, and *W* is the target size. Figure 1B shows the five combinations of targets’ size and distance, as well as the corresponding ID for this experiment. Note that each trial corresponded to one ID.

Participants had to actually point (actual or overt action) or to imagine themselves pointing (mental or covert action) between the targets as accurate and as fast as possible (i.e., adapted Fitts’s law motor paradigm, see also Maruff et al. (1999) and Gueugneau et al. (2008), while holding a pencil in their right-dominant hand. Actual and mental trials were performed with eyes open. Before an actual or mental trial, the participants placed the pencil in the center of one of the two targets (pseudorandom order, 50% right target and 50% left target). They were free to start the actual or mental movements when they felt ready. Relatively, long trial durations are necessary to obtain reliable measurements in mental movement simulation protocols because movement durations have a coarse resolution (Sirigu et al., 1996; Gentili et al., 2010; Demougeot and Papaxanthis, 2011). Therefore, one actual or mental trial consisted of five cyclical pointing movements between the targets, namely of 10 arm movements. For the mental trials, participants were requested to place their arm above the target, to keep it motionless during the whole trial, and to feel themselves performing the task (motor or first-person perspective) as they would actual do. Imagining a movement in the first person is a necessary condition to engage the motor system (Stinear et al., 2006; Gueugneau et al., 2013). For each actual trial, we measured the spatial precision of the pointing movements. Participants were informed that if they missed more than two targets during a trial, this one will be canceled (invalid trial) and retaken at the end of the session. Very few trials were repeated in both groups (<5%). Each trial was performed on a distinct sheet. The main experiment was preceded by a number of practice trials, which allowed participants to familiarize themselves with the task. The targets used in the practice session had different sizes (2 cm× 2 cm) from those used in the experiment. After this practice phase, all participants verbally reported being able to generate motor images after having practised 6–10 times. During the experiment, all participants performed eight actual and eight mental trials for each ID (80 trials per participant) in a pseudorandom order. When participants performed eight consecutive trials, they rested for ~1 min in order to prevent physical or mental fatigue. After the achievement of the experimental protocol, none of the participants reported mental or muscle fatigue and any difficulty to internally simulate the movements.

### Recording of movement time and statistical analysis

Actual and mental movement times were recorded by means of an electronic stopwatch hold by the participant in their left hand. They started the stopwatch when they actually or mentally initiated the movement and stop it when they finished pointing. We required the participants to record their actual and mental movement durations because they reportedly felt more comfortable manipulating the stopwatch themselves [see Skoura et al. (2008) and Personnier et al. (2010a)].

For each participant, the mean duration of movements and its SD was calculated over all trials. We first checked that all variables were normally distributed (*Shapiro-Wilk W test*; *P* > 0.05) and that their variance was equivalent (*Levene’s test*; *P* > 0.05). Then, we used three steps in our statistical analysis:

(i) First, we made a general analysis to investigate whether actual and mental movement times differed between groups. In this analysis, we did not consider movement times for each ID separately; instead, we averaged for each participant the times of the five ID. Using these average values, we performed an analysis of variance (ANOVA), with *group* (MDD patients, controls) as a between-subject factor and *movement* (actual, mental) as within-subject factors.
(ii) Then, we tested whether movement time was modulated as a function of ID. We performed ANOVA with *ID* (4.3, 4.9, 5.3, 5.9, and 6.3) as within-subject factor, for each independent variable separately (i.e., control-actual, control-mental, MDD-actual MDD-mental). We also performed a regression analysis between movement time and ID to verify their linear relationship as predicted by Fitts’s law. *R*^2^ values were compared by means of ANOVA with *group* as a between-subject factor and *movement* as within-subject factors.
(iii) Lastly, we compared the temporal similarities between actual and mental movements to appreciate to what extent action representation is similar to action production. When mental time significantly differs from actual time, one could argue that some aspects of movement production are not included, or partially integrated, into action representation. For that purpose, we calculated, for each participant, the index of mental performance (iMP):
  $$\mathrm{iMP}=\frac{D_{A}-D_{M}}{D_{A}}\times 100$$
  For each participant, iMP is defined as the absolute difference between the average time of actual movements (*D*_A_ in the formula; *n* = 8) and the average time of mental movements (*D*_M_ in the formula; *n* = 8). In order to account for inter-individual differences in movement duration, we divided this value by the average actual movement time (*D*_A_). An iMP value near to zero would indicate excellent mental performance; i.e., almost similar actual and mental movement durations. On the contrary, an index of 100% would indicate that the duration of mental movements is two times greater from that of actual movements. We averaged the absolute difference for the five ID and performed *independent t*-tests between the two groups. Statistical significance was accepted at *P* < 0.05 and *post hoc* differences were assessed by means of *Scheffé* test.

## Results

### Slower actual and mental movements for the depressive group

**Figure 2:**
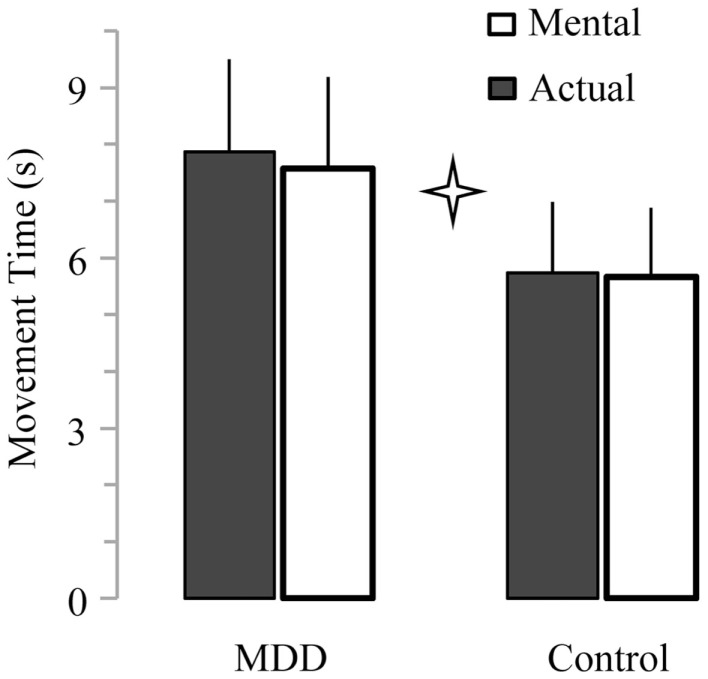
**Average (±SD) values of actual and mental movement times for both groups**. Star indicates significant differences between groups (*P* < 0.001).

Average times of actual movements ranged between 3.6 and 9.5 s for the control group and between 4.5 and 12.0 s for the MDD group. Average times of mental movements ranged between 3.3 and 9.1 s for the control group and between 4.7 and 11.7 s for the MDD group. Figure 2 shows average (+SD) actual and mental movement times (all ID mixed) for both groups. It is evident that patients with MDD performed the task slower (27.4 ± 2.9% for actual movements; 25.4 ± 4.1% for mental movements) than the participants of the control group (main effect of *group*; *F*_1,26_ = 18.33, *P* < 0.001). We did not find a main effect of *movement* (*P* = 0.22), or an interaction effect between *group* and *movement* (*P* = 0.42).

### Group difference in the modulation of mental movement times with the item difficulty

**Figure 3:**
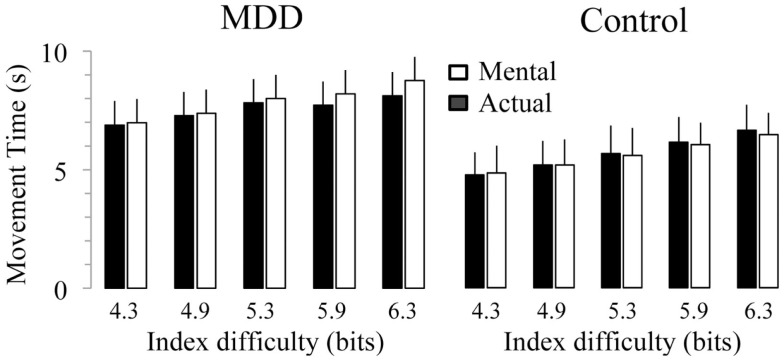
**Average (±SD) values of actual and mental movement times according to the index of difficulty (ID) for both groups**. Star indicates significant differences between groups (*P* < 0.001).

**Figure 4:**
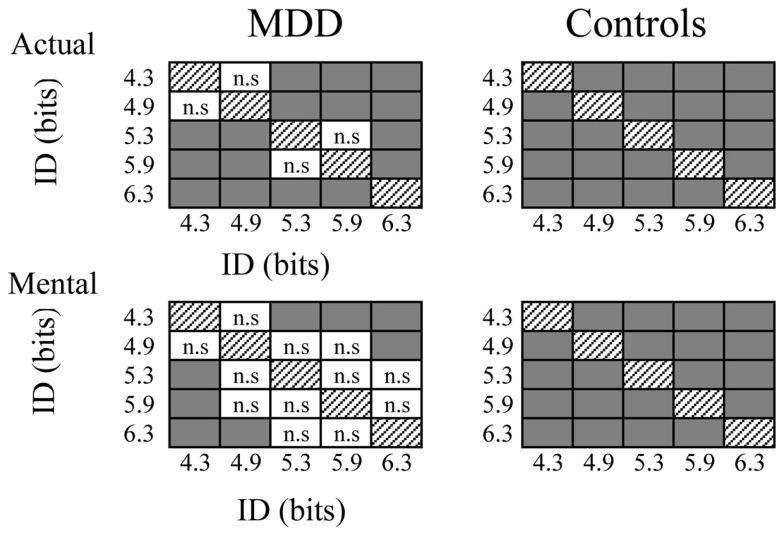
**Schematic representation of *post hoc* analysis regarding the influence of the index of difficulty on mental and actual movement times of both groups**. Gray squares indicate significant differences (*P* > 0.05), whereas white squares with indicate non-significant (n.s.) differences.

Figure 3 shows average durations (+SD) of actual and mental movements for the two groups and the five ID. For the control group, times of actual (*F*_4,52_ = 77.57, *P* < 0.0001) and mental (*F*_4,52_ = 45.89, *P* < 0.0001) movements progressively increased as the ID gradually increased. *Post hoc* comparisons (see Figure 4) showed that actual and mental movement times significantly differed between all ID (in all cases, *P* < 0.02). For the MDD group, there was also an effect for actual (*F*_4,52_ = 38.69, *P* < 0.0001) and mental (*F*_4,52_ = 7.21, *P* < 0.0001) movements. However, time modulation with ID in depressive patients was not as strong as that observed in the control group. For mental movement times, *post hoc* comparisons showed significant differences between ID_4.3_ versus ID_5.3_, ID_5.9_, and ID_6.3_ (in all cases, *P* < 0.02), and for the ID_4.9_ versus the ID_6.3_ (*P* = 0.01); for all the other comparisons, *P* > 0.1. For actual movement times, significant differences existed between several ID (in all cases, *P* < 0.05), except for the ID_4.3_ versus the ID_4.9_ (*P* = 0.10) and the ID_5.3_ versus the ID_5.9_ (*P* = 0.68).

The previous findings were further explored by performing a linear regression analysis between movement time and ID (see Table 2). ANOVA revealed a significant interaction effect between *group* and *movement type* for *R*^2^ values (*F*_1,26_ = 6.63, *P* = 0.016). *Post hoc* analysis showed that *R*^2^ values significantly differed between actual and mental movements for the MDD group (*P* < 0.001) but not for the control group (*P* = 0.28). Furthermore, *R*^2^ values significantly differed between groups for the mental (*P* < 0.001) but not for the actual movements (*P* = 0.50). We found similar results for the slope values (interaction effect between *group* and *movement type*; *F*_1,26_ = 5.35, *P* = 0.03). *Post hoc* analysis showed that slope values significantly differed between actual and mental movements for the MDD group (*P* < 0.001) but not for the control group (*P* = 0.82). Furthermore, slope values significantly differed between groups for the mental movements (*P* = 0.02) but not for the actual movements (*P* = 0.93).

**Table 2:** **Linear regression equations calculated between the index of difficulty and the average actual or mental movement durations for both groups**.

Group	Movement	Linear	*R*
MDD	Actual	*y* = 0.87*x* + 3.23	0,86
	Mental	*y* = 0.58*x* + 4.49	0,59
Control	Actual	*y* = 0.94*x* + 0.68	0,96
	Mental	*y* = 0.82*x* + 1.26	0,90

*MDD, major depressive disorder*.

### Group differences in mental performance

The temporal discrepancy between actual and mental movements is twice greater in the MDD group than in the control group (*t* = 2.38; *P* = 0.02). This temporal difference is further visible when the average times of actual movements are plotted across the average times of mental movements. It appears that scatterplot points are more spread in the MDD group than the control group. The correlation between actual and mental movement durations for the control group (*R*^2^ = 0.87) was significantly higher (*P* < 0.0001) than the correlation for the MDD group (*R*^2^ = 0.55).

### Relationships between actual movements, mental movements, and clinical factors

Correlational analyses were performed to examine the relationship between actual and mental movement times (all ID mixed) and clinically relevant variables including severity of depression. No significant correlations were found between MADRS, HDRS, SRRS, BDI scores and actual (in all comparisons: 0.01 < *R*^2^ < 0.15; −0.06 < *t* < 1.20; 0.26 < *P* < 0.94) or mental movement times (0 < *R*^2^ < 0.14; −0.07 < *t* < 1.14; 0.29 < *P* < 0.94).

## Discussion

The present study investigated actual movement production and motor imagery ability in patients with severe unipolar major depression. Motor imagery was evaluated by means of the mental chronometry paradigm in an arm pointing motor task with increasing spatiotemporal constraints (i.e., speed/accuracy trade-off motor paradigm). Our main findings revealed a global slowness of both actual and mental movements and a decline in the ability to mentally represent actions in patients with MDD compared with a control group of healthy adults. These results suggest that unipolar depression significantly affects the higher stages of action planning with depression.

### Alteration of actual and mental movements in unipolar depression

The results of our study indicate that patients with MDD produced pointing arm movements significantly slower than healthy adults. This observation corroborates and expands previously described motor deficits in psychomotor function in unipolar depression. Notably, depressed patients exhibit overt signs associated with a global slowness, such as reduced movement velocities, increased reaction times, and increased movement times (Sabbe et al., 1999). This motor slowness has been mainly related to the dopamine dysregulation in depression (Schrijvers et al., 2008). Here, it is of interest that, despite this general slowness, patients modulated their actual movement time according to both target size and target distance; i.e., with respect to the task difficulty as preconized by Fitts’s law. At a first glance, this may suggest that the higher levels of movement organization remains intact in MDD patients, because a motor law (here, the Fitts’s law) is conserved, and that motor slowness is perhaps due to deficits at the lower stages of movement production, such as execution and sensorimotor control.

However, our findings regarding mental movements seem to not support this premise. One of the most prominent results of our study was that duration of imagined movements, as those of actual movement, was significantly longer in depressed patients compared with healthy participants. As during motor imagery no actual movement occurs, this finding denotes that motor impairments in unipolar depression have to a great extent a central origin, instead of peripheral deficiencies. This idea is further supported by the greater temporal discrepancies between actual and mental movements in depressed patients. These observations point out a decline in the capacity to mentally representing actual movement production. In other words, action planning and representation in depressed patients do not correspond to actual movement production; that is, the ongoing movement is not executed as represented. More appealing was the finding regarding the respect of Fitts’s law during mental movements. Our experiment revealed that depressed patients modulated, to some extent, mental movement durations according to the difficulty of the task. However, this modulation was not as strong as that of healthy subjects. For instance, the slope and correlation values of the relationship between mental movements and task difficulty significantly differed between the two groups (see Table 2). Together, our findings regarding mental movement simulation indicate an alteration in action representation and planning in depressed patients. This extends the results of previous studies highlighting impairment of mental transformation abilities in major depressive disorder (Rogers et al., 2000; Chen et al., 2013). In fact, compared to controls, MDD patients exhibit longer reaction times during mental rotation tasks and experience progressively greater slowing as a function of the angle of rotation, as a reflect of specific deficits of visuospatial cognitive operation (Chen et al., 2013). Comparing internally and externally cued response selection and initiation, several authors demonstrated that depressed patients were particularly slowed when movements involved internal movement selection (Rogers et al., 1987, 2000; Hoffstaedter et al., 2012). Collectively, these results suggest that depression influences the generation and manipulation of intended actions. Current results enlarge those of previous studies, which have used several motor imagery paradigms to gain insight into the action system of diseased populations.

### Forward internal models for action in major depression

Forward internal models mimic the causal flow of the physical process by predicting the consequences (e.g., position, velocity) of a motor command (Wolpert and Miall, 1996; Desmurget and Grafton, 2000; Wolpert and Flanagan, 2001). The use of a forward internal model could explain the temporal equivalence between actual and mental arm movements in healthy subjects (Pozzo et al., 2006; Papaxanthis et al., 2012; Michel et al., 2013). During motor imagery, timing information for the simulated movement is provided by the forward internal model, which on the basis of the correctly prepared (efferent copy is available), but blocked neural commands (note that no actual movement occurs during motor imagery), predicts the future sensorimotor state of the movement. Therefore, accurate forward models can provide similar temporal estimations, i.e., isochrony, during both actual and mental movements under varying spatiotemporal constraints. Interestingly, our results showed that depressed patients did not fully integrate task constraints (i.e., target size and movement speed) during the mental simulation process. In particular, greater absolute differences between actual and mental movements were observed in patients than health participants. This may be due to the fact that forward internal models, used in mental prediction of motor actions, are not well preserved in major depressive disorder.

These behavioral findings may reflect functional and structural brain changes of a network associated with action representation. Specific deficits of motor control in depression have been attributed to disturbances in the higher cognitive control centers, including the dorsolateral prefrontal cortex, the parietal cortex the anterior cingulate cortex, and the basal ganglia (Walther et al., 2012). Due to the involvement of these areas in motor imagery process, these dysfunctions could underlie the alteration of the temporal processing of imagined actions in depression. Recent findings seem to point in that direction. Liberg and collaborators found that activation in the brain areas involved in motor selection, planning, and preparation was altered in patients with bipolar depression (Liberg et al., 2013). Precisely, during motor imagery, patients with bipolar depression activated the posterior medial parietal cortex, the posterior cingulate cortex, the premotor cortex, the prefrontal cortex, and the frontal poles more than the controls did.

Our results must be viewed with caution as they need further investigation and generalization. The effect of severity and state of depression needs to be analyzed in longitudinal studies including neuropsychiatric control groups and depressive subgroups. Preoccupation with precision is an important confounding variable to consider, which could impact accuracy and anxiety levels in the subjects (Sobin and Sackeim, 1997). Motivational factors including interest, pleasure, and reactivity to pleasurable stimuli contribute to the initiation and progression of motor activity, and may interfere with the expression of behaviors (Lemke et al., 1999; Scheurich et al., 2008). Recent studies have highlighted the importance of deficits in willingness to expend effort for reward in depressed patients. Reward-related deficits, a crucial aspect of anhedonic symptoms, might involve a failure to integrate cost/benefit information in a consistent manner (Treadway et al., 2012; Yang et al., 2014). This hypothesis is supported by previous report on the role of dopamine in computing cost-benefit computations and in modulating the amount of effort allocated to obtain rewarding outcomes (Der-Avakian and Markou, 2012; Salamone and Correa, 2012). Effort-related motivational impairments and observable psychomotor alterations may have common underlying neurobiological mechanisms involving dopaminergic pathways in mesolimbic structures (Salamone and Correa, 2012; Treadway and Zald, 2013). Therefore, future investigations combining laboratory effort-tasks and motor imagery paradigms may provide further insights into the neurobiological process underlying the inhibition of activity in mood disorders.

## Conclusion

Motor imagery studies in normal adults indicated that the same motor representation governs an action whether it is executed or imagined, and time constraints operate in the same way in both modalities of action. Our patients’ impaired performances suggest that mental prediction of motor actions is not preserved in MDD, with specific alterations of the higher stages of action planning. A decline in cognitive processing associated with the ability to mentally represent actions leads to difficulties making accurate predictions of intended actions. The alteration of this mechanism is a relevant finding for physical and cognitive interventions in depression. Moreover, this paradigm offers an innovative approach for the study of motor and cognitive components of PMR in depression and might provide objective parameters to measure antidepressant response.

## Conflict of Interest Statement

The authors declare that the research was conducted in the absence of any commercial or financial relationships that could be construed as a potential conflict of interest.
